# Molecular signatures of maturing dendritic cells: implications for testing the quality of dendritic cell therapies

**DOI:** 10.1186/1479-5876-8-4

**Published:** 2010-01-15

**Authors:** Ping Jin, Tae Hee Han, Jiaqiang Ren, Stefanie Saunders, Ena Wang, Francesco M Marincola, David F Stroncek

**Affiliations:** 1 Department of Transfusion Medicine, Clinical Center, National Institutes of Health, Bethesda, Maryland, USA; 2 Department of Laboratory Medicine, Inje University Sanggye Paik Hospital, Seoul, Korea

## Abstract

**Background:**

Dendritic cells (DCs) are often produced by granulocyte-macrophage colony-stimulating factor (GM-CSF) and interleukin-4 (IL-4) stimulation of monocytes. To improve the effectiveness of DC adoptive immune cancer therapy, many different agents have been used to mature DCs. We analyzed the kinetics of DC maturation by lipopolysaccharide (LPS) and interferon-γ (IFN-γ) induction in order to characterize the usefulness of mature DCs (mDCs) for immune therapy and to identify biomarkers for assessing the quality of mDCs.

**Methods:**

Peripheral blood mononuclear cells were collected from 6 healthy subjects by apheresis, monocytes were isolated by elutriation, and immature DCs (iDCs) were produced by 3 days of culture with GM-CSF and IL-4. The iDCs were sampled after 4, 8 and 24 hours in culture with LPS and IFN-γ and were then assessed by flow cytometry, ELISA, and global gene and microRNA (miRNA) expression analysis.

**Results:**

After 24 hours of LPS and IFN-γ stimulation, DC surface expression of CD80, CD83, CD86, and HLA Class II antigens were up-regulated. Th1 attractant genes such as CXCL9, CXCL10, CXCL11 and CCL5 were up-regulated during maturation but not Treg attractants such as CCL22 and CXCL12. The expression of classical mDC biomarker genes CD83, CCR7, CCL5, CCL8, SOD2, MT2A, OASL, GBP1 and HES4 were up-regulated throughout maturation while MTIB, MTIE, MTIG, MTIH, GADD45A and LAMP3 were only up-regulated late in maturation. The expression of miR-155 was up-regulated 8-fold in mDCs.

**Conclusion:**

DCs, matured with LPS and IFN-γ, were characterized by increased levels of Th1 attractants as opposed to Treg attractants and may be particularly effective for adoptive immune cancer therapy.

## Introduction

Dendritic cells (DC) are key players in both innate and adaptive immune responses. They are potent antigen presenting cells that recognize, process, and present antigens to T-cells *in vivo *[1-3]. Consequently, DC-based immunotherapy has become one of the most promising approaches for the treatment of cancer [4,5]. The frequency of DCs in the peripheral blood is naturally low and they are difficult to separate from other peripheral blood leukocytes [6], therefore, to enhance DC function, hematopoietic progenitor cells or peripheral blood monocytes are usually used to produce mDC *in vitro *by culture with growth factors and cytokines [6,7].

Large quantities of mononuclear cells can easily be collected from the peripheral blood by leukapheresis. Monocytes can be isolated from other leukocytes collected by apheresis with high purity by adherence, elutriation, or using immunomagnetic beads [8-10]. To produce immature DCs (iDCs), monocytes are usually incubated with granulocyte-macrophage colony-stimulating factor (GM-CSF) and interleukin-4 (IL-4). Because mature DCs (mDCs) are superior to iDCs for the stimulation of cytotoxic T-cells, iDCs derived from monocytes are often treated with various exogenous stimuli known to induce DCs maturation including lipopolysaccharide (LPS) and interferon-γ (IFN-γ) [5,11]. One of the goals of this study was to characterize the molecular profile of changes associated with LPS and IFN-γ induced DC maturation to estimate the effectiveness of these mDCs in adoptive immune cancer therapy.

When developing cellular therapies such as mDCs it is often necessary to compare products manufactured with a standard method and an alternative method. It is also necessary to determine if products manufactured from the starting material of different people are consistent or similar. Once the manufacturing process has been established and clinical products are being manufactured, clinical cellular therapies must also be assessed for potency. Another goal of this study was to identify molecular biomarkers that were associated with DC maturation and in order to characterize mDCs and that could be used for consistency, comparibility, and potency testing.

DCs are often assessed by flow cytometry for the expression of the costimulatory molecules CD80 and CD86, the maturation marker CD83, the chemokine receptor CCR7, and antigen presentation molecules, HLA class II antigens, to document the transition of iDCs to mDCs. Some cellular therapy laboratories also test the function of DCs by measuring their ability to produce IL-12, IL-10, IL-23 or IFN-γ following stimulation. However, the diverse functions of DC therapies indicate that additional biomarkers are necessory to characterize mDCs. Based on the multiple functions of DCs and their broad spectrum of effector molecules, it is highly improbable that a limited number of biomarkers can adequately measure DC potency. But whole transcriptome expression analysis and microRNA (miR) profiling analysis of the DC maturation process could provide better insight into DC biology and identify biomarkers that are indicators of DC potency.

Although monocytes, iDCs, and mDCs have been characterized at a molecular level, few studies have comprehensively studied the molecular events associated with DC maturation. In this study we compared the kinetics of global changes of both gene and miR expression associated with LPS and IFN-γ induced DC maturation. Gene and miR changes in DCs were assessed after 4, 8 and 24 hours of LPS and IFN-γ stimulation. To validate the functional activity of DCs, we also tested soluble protein production in culture supernatant after 24 hours of maturation and after incubation with CD40 ligand transfected mouse fibroblasts.

## Materials and methods

### Study design

Peripheral blood mononuclear cell (PBMC) concentrates were collected using a CS3000 Plus blood cell separator (Baxter Healthcare Corp., Fenwal Division, Deerfield, IL) from 6 healthy donors in the Department of Transfusion Medicine (DTM), Clinical Center, National Institutes of Health (NIH). All donors signed an informed consent approved by a NIH Institutional Review Board. Monocytes were isolated from the PBMC concentrates on the day of PBMC collection by elutriation (Elutra^®^, Gambro BCT, Lakewood, CO) using the instrument’s automatic mode according to the manufacturer's recommendations. The monocytes were treated with GM-CSF (2000 IU/mL, R&D Systems, Minneapolis, MN) and IL-4 (2000 IU/mL, R&D Systems) for 3 days to produce iDCs. The iDCs were then treated for 24 hours with LPS and IFN-γ to produce mDCs. The results of analysis of iDCs and mDCs by flow cytometry and gene expression profiling have been previously published [12].

### DC preparation, maturation, and harvest

The elutriated monocytes from each donor were suspended at 6.7 × 10^6^/mL with RPMI 1640 (Invitrogen, Carlsbad, CA) supplemented with 10% fetal calf serum (FSC) (Invitrogen), 2 mM L-glutamine (Invitrogen), 1% nonessential amino acids (Invitrogen), 1% pyruvate (Invitrogen), 100 units/mL penicillin/streptomycin (Invitrogen), and 50 μM 2-mercaptoethanol (Sigma, St Louis, MO). A total of 10 mL of monocyte suspension was cultured in T25 culture flasks (Nalge Nunc International, Rochester, NY) overnight in a humidified incubator with 5% CO_2 _at 37°C. On Day 1, 2000 IU/mL human IL-4 (R&D Systems) and 2000 IU/mL GM-CSF (R&D Systems) were added to the culture. On Day 3, an additional 2000 IU/mL IL-4 and GM-CSF were added. To induce DC maturation, on day 4, 100 ng/mL LPS (Sigma) and 1000 IU/mL IFN-γ (R&D Systems) were added. The DCs were harvested at 0, 4, 8 and 24 hours (h) after the addition of LPS and IFN-γ. To remove the adherent DCs, 2 mM EDTA-PBS was added to each flask on ice. The harvested cells were pelleted, washed twice with HBSS, and resuspended in RPMI 1640. The total number of cells harvested and their viability was measured microscopically after adding Trypan Blue.

### Flow cytometeric analysis

The purity of the elutriated monocytes was evaluated by flow cytometry using CD14-PE, CD19-FITC, CD3-PE-Cy5, and CD56-APC (Becton Dickinson, Mountain View, CA) and isotype controls (Becton Dickinson). To confirm the maturation of the DCs, the harvested DCs were tested with CD80-FITC, CD83-PE, CD86-FITC, HLA-DR-PE-Cy5, and CD14-APC (Becton Dickinson) and isotype controls (Becton Dickson). Flow cytometry acquisition and analysis were performed with a FACScan using CellQuest software (Becton Dickinson).

### Analysis of DC function and cytokine generation

To measure DC cytokine production, iDC and mDCs (100,000 cells/ml) were co-incubated with 50,000 cells/ml of adherent mouse fibroblasts transfected to express human CD40-Ligand (CD40L-LTK) in 48-well plates. This cell line was kindly provided by Dr. Kurlander (Department of Laboratory Medicine, Clinical Center, National Institutes of Health, Bethesda, MD). Before (0 hour) and after 24 hours of stimulation the supernatant was collected and the samples were analyzed by protein expression profiling. The levels of 50 soluble factors were assessed on an ELISA-based platform consisting of multiplexed assays that measured up to 16 proteins per well in standard 96 well plates (Pierce Search Light Proteome Array, Boston, MA)[13].

### RNA preparation, amplification, and labeling for oligonucleotide microarray analysis

Total RNA was extracted from the DCs using Trizol (Invitrogen, Carlsbad, CA). RNA integrity was assessed using an Agilent 2100 Bioanalyser (Agilent Technologies, Waldbronn, Germany). Total RNA (3 μg) from the DCs was amplified into anti-sense RNA (aRNA). While total RNA from PBMCs pooled from the 6 normal donors was extracted and amplified into aRNA to serve as the reference. Pooled reference and test aRNA were isolated and amplified using identical conditions and the same amplification/hybridization procedures to avoid possible interexperimental biases. Both reference and test aRNA were directly labeled using ULS aRNA Fluorescent Labeling kit (Kreatech, Amsterdam, Netherlands) with Cy3 for reference and Cy5 for test samples.

Human oligonucleotide microarrays spanning the entire genome were printed in the Infectious Disease and Immunogenetics Section, DTM, Clinical Center, NIH using a commercial probe set containing 35,035 oligonucleotide probes, representing approximately 25,100 unique genes and 39,600 transcripts excluding control oligonucleotides (Operon Human Genome Array-Ready Oligo Set version 4.0, Huntsville, AL, USA). The design of the probe set was based on the Ensemble Human Database build (NCBI-35c), with full coverage of the NCBI human Reference sequence dataset (April 2, 2005). The microarray was composed of 48 blocks with one spot printed per probe per slide. Hybridization was carried out in a water bath at 42°C for 18 to 24 hours and the arrays were then washed and scanned on a GenePix scanner Pro 4.0 (Axon, Sunnyvale, CA) with a variable photomultiplier tube to obtain optimized signal intensities with minimum (<1% spots) intensity saturation.

### miR expression analysis

A miRNA probe set was designed using mature antisense miRNA sequences (Sanger data base, version 9.1) consisting of 827 human, mouse, rat and virus probes plus two control probes. The probes were 5' amine modified and printed in duplicate in the Immunogenetics Section of the DTM on CodeLink activated slides (General Electric, GE Health, NJ, USA) via covalent bonding. 3 μg total RNA was directly labeled with miRCURY™ LNA Array Power Labeling Kit (Exiqon) according to manufacturer's procedure. The total RNA from Epstein-Barr virus (EBV)-transformed lymphoblastoid cell line was used as the reference for the miRNA expression assay. The test samples were labeled with Hy5 and the references with Hy3. After labeling, both the sample and the reference were co-hybridized to the miRNA array at room temperature overnight and the slides were washed and scanned by GenePix scanner Pro 4.0 (Axon, Sunnyvale, CA, USA).

### Data processing and statistical analyses

The raw data set was filtered according to a standard procedure to exclude spots below a minimum intensity that arbitrarily was set to an intensity parameter of 200 for the oligonucleotide arrays and 100 for the miR arrays in both fluorescence channels. If the fluorescence intensity of one channel was great than 200 for oligonuceotide array (100 for miR array), but the other was below 200(100), the fluorescence of the low intensity channel was arbitrarily set to 200(100). Spots with diameters <20 μm from oligonucleotide arrays, <10 μm from microRNA arrays and flagged spots were also excluded from the analysis. The filtered data was then normalized using the median over the entire array and retrieved by the BRB-ArrayTools http://linus.nci.nih.gov/BRB-ArrayTools.html which was developed at the National Cancer Institute (NCI), Biometric Research Branch, Division of Cancer Treatment and Diagnosis. Hierarchical cluster analysis and TreeView software were used for visualization of the data [14,15]. Gene annotation and functional pathway analysis was based on the Database for Annotation, Visualization and Integrated Discovery (DAVID) 2007 software [16] and GeneCards website http://www.genecards.org/index.shtml.

### miR and gene expression analysis by quantitative PCR

To validate the results of the microarray analysis, three miR and 4 genes were selected for analysis by quantitive real-time/reverse-transcription polymerase chain reaction (RT-PCR). miR expression was measured and quantified by TaqMan MicroRNA Assays (Applied Biosystems, Foster City, CA). Quantitative RT-PCR for miR-146a, miR-146b, and miR-155 were performed according to the manufacturer's protocol and normalized by RNU48 (Applied Biosystems). Gene expressions for HLA-DRA (Assay ID Hs00219578_m1), HLA-DRB1 (Assay ID Hs99999917_m1), CCR7 (Assay ID Hs99999080_m1), and CD86 (Assay ID Hs00199349_m1) were quantified by TaqMan Gene Expression Assays (Applied Biosystems) according to manufacturers' protocol and normalized by GAPDH (Assay ID Hs99999905_m1). Differences in expression were determined by the relative quantification method; the Ct values of the test genes were normalized to the Ct values of endogenous control GAPDH. The fold change or the relative quantity (RQ) was calculated based on RQ = 2^-ΔCt^, where ΔCt = average Ct of test sample - average Ct of endogenous control sample.

## Results

### Changes in DC antigen expression

Immature DCs were produced from peripheral blood monocytes from 6 healthy subjects by stimulation with GM-CSF and IL-4 for 3 days. The iDCs were further stimulated with LPS and IFN-γ and the expression of surface markers CD80, CD83, CD86, and HLA-DR were analyzed by flow cytometry before and after 4, 8, and 24 hours of LPS and IFN-γ stimulation. The expression of all 4 antigens increased during maturation (Table 1).

**Table 1:** Comparison of DC expression of CD14, CD80, CD83, CD86, and HLA-DR antigens according to maturation time

	Percent of DCs expressing each antigen*					
Maturation Time	CD80	CD83	CD86	CD83 & CD86	HLA-DR	CD14
0 h	29.2 ± 9.5	36.6 ± 11.9	26.0 ± 13.2	20.8 ± 14.5	80.6 ± 10.3	0.22 ± 0.11
4 h	47.6 ± 16.9	67.4 ± 14.6	82.8 ± 6.3	69.0 ± 7.8	93.7 ± 3.6	0.18 ± 0.17
8 h	79.3 ± 12.7	80.0 ± 11.5	90.9 ± 6.2	81.6 ± 13.3	95.6 ± 2.2	0.19 ± 0.14
24 h	89.6 ± 7.5	93.8 ± 6.3	96.7 ± 1.8	97.8 ± 0.6	98.2 ± 1.1	0.10 ± 0.07

*Values represent the mean ± 1 standard deviation

h = hours

### Kinetics of the gene expression changes during DC maturation

**Figure 1:**
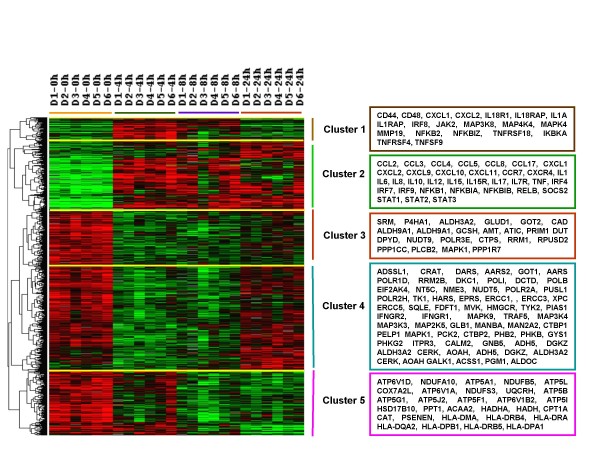
**Gene expression changes in maturing DCs**. Immature DCs from 6 healthy subjects were incubated with LPS and IFN-γ. After 0, 4, 8, and 24 hours of culture, DCs were analyzed by gene expression profiling using a microarray with 35,035 oligonucleotide probes. The 2,370 differentially expressed genes (F-test: p < 0.001) were analyzed by supervised hierachical clustering. Immature DCs are indicated by the orange bar, iDCs cultured with LPS and IL-4 for 4 hours by the green bar, 8 hours by the purple bar, and 24 hours by the red bar. The genes sorted into 5 separate clusters and representive genes from each of the 5 clusters are shown.

Global gene expression was assessed in DCs from the 6 subjects pre-treatment (time 0, iDCs) and after 4, 8 and 24 hours of LPS and IFN-γ stimulation. A total of 2,370 genes differed significantly among the maturation time groups (F-test; p < 0.001). Supervised hierarchical clustering revealed distinct clusters of genes that characterized each of the maturation times (Figure 1). Genes in clusters 1 and 2 were up-regulated during maturation and those in clusters 3, 4, and 5 were down-regulated. At hours 4 and 8, genes in cluster 1 were up-regulated compared to iDCs but returned to base levels after 24 hours. Cluster 2 genes were up-regulated on hours 4 through 24 of maturation. Cluster 3 and 4 genes were down-regulated on hours 4 and 8 but then returned to baseline levels after 24 hours. However the level of expression of genes in cluster 4 was greater after 24 hours than those in cluster 3. After 4 hours the expression of genes in cluster 5 were similar to baseline levels, but were then down-regulated on hours 8 and 24.

Canonical pathway analysis showed that genes in each of these 5 clusters belong to different pathways (See additional file 1, table S1). Genes in Clusters 1 and 2 were most likely to be in pathways involved with the cellular immune response (See additional file 1, table S1, bold and *), cytokine signaling (See additional file 1, table S1, italics and #), transcriptional regulation and the inflammatory response. This is consistent with cells that are ready to respond or are already responding to external stimuli. In contrast, genes in Clusters 3 and 4 were most likely to belong to pathways involved with metabolism (See additional file 1, table S1, bold and †). Genes in Cluster 5 also belonged to metabolism pathways as well as Humoral Immune Response and Pathogen-Influenced Signaling Pathways (See additional file 1, table S1, italics and $). The specific genes that were differentially expressed among the DCs stimulated with LPS and IFN-γ for different durations of time and their fold-changes are summarized in Tables S2 and S3 [see Additional Files 2 and 3] (t-test, p ≤ 0.001 compared to hr 0).

The genes up-regulated during DC maturation included many involved with immune function, cell differentiation, and migration. Several chemokines and their ligands were up-regulated during maturation. For example CCR7, which enhances the ability of DCs to migrate to lymphoid nodes was markedly up-regulated during maturation. Its expression was increased more than 10-fold at all times during maturation and was greatest after 24 hours of maturation (up-regulated 18-fold). Moreover, the expression of Oncostatin M (OSM), which enhances the expression of the CCR7 ligand CCL21 by microvascular endothelial cells and increases the efficiency of dendritic cell trafficking to lymph nodes [17], was increased 5- to 6-fold during maturation. In addition, CXCR4, a chemokine receptor involved with DC migration to lymphoid nodes, was up-regulated 3-fold after 24 hours of maturation [18]. However, the expression of several inflammatory chemokine receptors including CCR1 and CCR2 fell during maturation.

The expression of inflammatory chemokine ligands including CCL2 (MCP-1), CCL3 (MIP1α), CCL4 (MIP1β), CXCL1 (GROα) and CXCL9, reached a peak at 4 hours of maturation but then rapidly returned to baseline levels. However, the expression of chemokines CCL5 (RANTES), CCL8 (MCP-2), and CXCL10 peaked after 8 hours and sustained high expression levels through 24 hours. Chemokine ligands that were part of Toll-like receptor signaling pathways, such as CCL3, CCL4, CCL5, CXCL9 (MIG), CXCL10 (IP-10), and CXCL11 (ITAC), were all up-regulated more than 7-fold during maturation. The levels of most of these genes peaked at hour 4 except for CCL5 and CXCL10 which peaked at hour 8 and sustained high levels of expression through hour 24. Chemokine ligands that preferentially attract Th1 T cells such as CXCL9, CXCL10, and CXCL11 were also markedly increased after 4 hours. However, two chemokine ligands for CCR4, which are important attractants of Th2 cells CCL17 (TARC) and CCL22 (MDC), were only slightly up-regulated or showed no significant change after 24 hours of maturation.

The expression of proinflamatory cytokines such as IL-1β (IL-1B), IL-6, IL-8, IL-15, and TNF were up-regulated more than 10-fold and their expression reached a peak after 4 hours. The expression of IL-12p40 (IL12B), IL-10 and IL-27 were up-regulated less than 10-fold after 4 hours of maturation and remained at the same level after 24 hours.

The costimulatory molecules, CD80 and CD86, and maturation marker CD83, all classic DC surface markers, were up-regulated durning DC maturation [see Additional File 2, Table S2]. The expression of all three was above baseline levels throughout maturation. The expression of CD83 was markedly increased, 17- to 23-fold, compared to 1.3- to 3.5-fold for CD80 and CD86.

Genes encoding the major histocompatibility complex (MHC) Class I molecules (HLA-A, B, C, F, G, and H), proteosome activator subunit 2 (PSME2), and antigen peptide transport 1-2 (TAP1, 2) which are important for antigen processing and presentation were all up-regulated more than 2-fold through the 24 hours of maturation(see additional file 2, table S2). Interestingly, MHC Class II genes were down-regulated during maturation, although analysis by flow cytometry showed that the expression cell surface HLA-DR protein increased during maturation (Table 1).

The transcription factor RelB, which is essential for the development and function of DCs, was up-regulated approximately 3-fold at 4 and 8 hours of maturation and 6-fold after 24 hours. This transcript factor directs the development of CD14+ monocytes to myeloid DCs rather than to macrophages. Another family of transcription factors which are involved in DC differentiation and function are the Interferon regulatory factors (IRFs). Two members of this family are especially important, IRF4 and IRF8, and both were up-regulated during DC maturation. SOCS1 (Suppressors of cytokine signaling 1) which has been shown to play a major role in regulation DC was increased 1.6-fold after 4 hours and SOCS2 expression was increased 4.8- to 5.7-fold throughout maturation(see additional file 2, table S2).

The expression of some genes was greatest in mDCs. Among these genes, two involved with antigen presentation, LAMP3 and MARCKSL1, were up-regulated 4 hours after LPS and IL-4 stimulation and their expression continued to increase throughout the study period. The maximum change in expression of LAMP3 and MARCKSL1 were observed after 24 hours of maturation with a 37-fold and 21-fold increase respectively. The expression of the cell cycle and cell signaling genes GADD45A and RGS1 also increased most after 24 hours of maturation. Their expression peaked with 50-fold and 28-fold up-regulation respectively(see additional file 2, table S2).

Genes that were down-regulated during DC maturation included CD1C, CD33 and CD14. CD14 was only down-regulated 1.5- to 2.0-fold during the 24 hour period, but CD33 was down-regulated 6- to 64-fold and CD1C was down-regulated 57- to 81-fold (see additional file 3, table S3).

**Figure 2:**
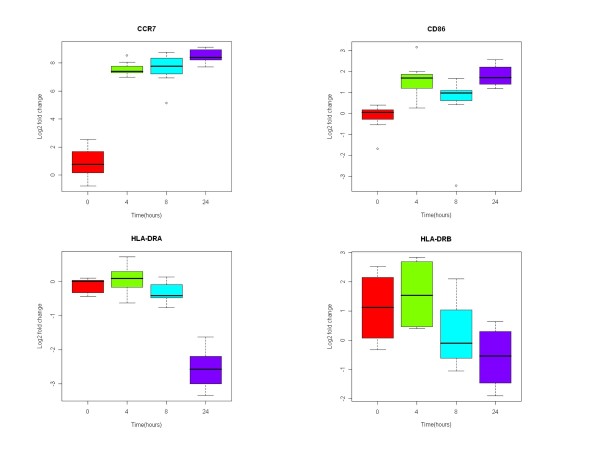
**Change in the expression of CCR7, CD86, HLA-DRA, and HLA-DRB during DC maturation**. iDCs from 6 healthy subjects sampled after 0, 4, 8 and 24 hours of culture in LPS and IFN-γ were analyzed by quantitive RT-PCR for the expression of CCR7, CD86, HLA-DRA, and HLA-DRB.

To validate the microarray results, 4 genes (HLA-DRA, HLA-DRB1, CCR7, and CD86) were selected for analysis by quantitive RT-PCR. HLA-DRA and HLA-DRB1 were selected because although the expression of HLA Class II antigens are increased in mDCs, microarray analysis found that the expression HLA-DRA and HLA-DRB1 were down-regulated. CCR7 and CD86 were selected because microarray analysis showed that the expression of both genes were up-regulated during DC maturation. In addition, CCD7 is an important chemotaxis receptor and CD86 and important costimulatory molecule. The results from quantitive RT-PCR were consistent from those obtained with the microarrays (Figure 2).

### miR expression during DC maturation

**Figure 3:**
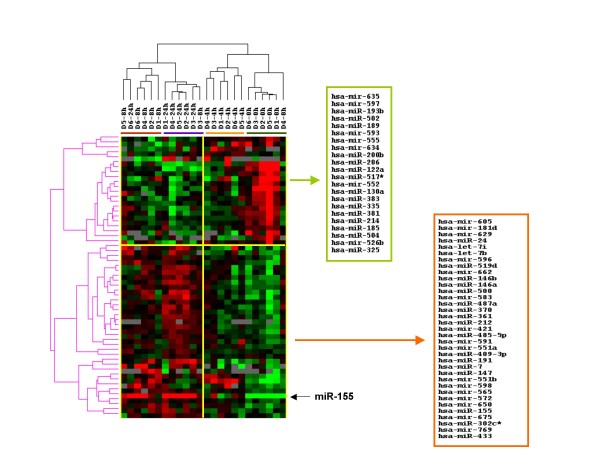
**miR expression changes in maturing DCs**. Immature DCs from 6 healthy subjects were incubated with LPS and IFN-γ and after 0, 4, 8, and 24 hours of culture, they were analyzed by global microRNA expression profiling using a microarray with 827 probes. The differentially expressed human miR (F-test: p < 0.05) were analyzed by supervised hierachical clustering. The samples clustered into 4 groups based on maturation time, iDC are indicated by the green bar, DCs cultured for 4 hours by the orange bar, DCs cultured for 8 hours by the red bar, and 24 hours by the purple bar. The miRs sorted into 2 separate clusters and miRs from each of the clusters are shown.

The expression of miR was also measured during DC maturation. Among the 474 miR analyzed 57 were differentially expressed (F-test, p ≤ 0.05) and were present in more than 80% of the samples. Hierarchical cluster analysis separated the samples into 2 major groups; an early group which included DCs samples treated with LPS and IFN-γ for 0 and 4 hours and a late group which containing DC samples treated with LPS and IFN-γ for 8 and 24 hours (Figure 3). Both the early and late groups contained two subgroups. The samples in these four subgroups were separated according to maturation time; hours 0, 4, 8 and 24. In contrast to gene expression, where several patterns or waves of expression were noted, only two general patterns were noted for miR analysis: miR whose expression decreased with maturation and miR whose expression increased with maturation. Compared with iDC, miR-155, miR-605, miR-146a, miR-146b, miR-623, miR-583, miR-26a, miR-519d, miR-126, and miR-7 were significantly up-regulated in mDC. miR-155 was up-regulated the most (8-fold) after 24 hours. The other miRs were up-regulated 1.5- to 1.76-fold. miR-375, miR-451, miR-593, miR-555, and miR-134 were down-regulated significantly (2.3- to 2.9-fold) after 24 hours (Table 2).

**Table 2:** MicroRNA (miRNA) whose expression changed in iDCs following LPS and IFN-γ stimulation (T-test, p ≤ 0.05)

Up-regulated MicroRNA				Down-regulated MicroRNA			
	
	Fold Increase				Fold Decrease		
			
MicroRNA	4 h	8 h	24 h	MicroRNA	4 h	8 h	24 h
hsa-miR-155	3.29	4.65	8.01	hsa-miR-375	-1.75	NS	-2.9
hsa-mir-605	NS	1.45	1.76	hsa-miR-451	-2.3	NS	-2.76
hsa-miR-146a	1.27	1.45	1.73	hsa-mir-593	-1.79	-1.85	-2.43
hsa-mir-623	5.59	NS	1.72	hsa-mir-555	-1.68	-1.82	-2.37
hsa-miR-146b	NS	1.43	1.7	hsa-miR-134	NS	-1.56	-2.29
hsa-mir-583	NS	NS	1.59	hsa-miR-200b	-1.57	-1.47	-1.83
hsa-miR-26a	NS	NS	1.58	hsa-miR-122a	-1.45	-1.63	-1.83
hsa-miR-519d	NS	NS	1.54	hsa-miR-452	-1.49	NS	-1.79
hsa-miR-126	NS	NS	1.53	hsa-miR-215	-1.4	NS	-1.72
hsa-miR-7	NS	1.35	1.51	hsa-mir-644	NS	NS	-1.7
hsa-let-7b	NS	1.29	1.49	hsa-miR-504	-1.73	-1.25	-1.67
hsa-miR-370	NS	1.19	1.48	hsa-miR-499	NS	NS	-1.65
hsa-let-7a	NS	1.35	1.48	hsa-miR-335	-1.32	NS	-1.61
hsa-let-7i	NS	1.2	1.48	hsa-mir-554	NS	NS	-1.59
hsa-miR-30a-3p	NS	NS	1.47	hsa-miR-422a	-1.37	-1.43	-1.58
hsa-mir-565	2.43	1.99	1.46	hsa-miR-383	-1.42	-1.45	-1.57
hsa-let-7e	NS	NS	1.46	hsa-miR-138	NS	NS	-1.54
hsa-mir-598	1.74	1.68	1.45	hsa-miR-206	-1.33	-1.38	-1.53
hsa-mir-594	NS	NS	1.44	hsa-mir-552	-1.35	-1.51	-1.53
hsa-let-7c	NS	NS	1.44	hsa-miR-325	NS	NS	-1.53
hsa-miR-212	NS	1.22	1.43	hsa-miR-517*	-1.46	-1.38	-1.5
hsa-let-7d	NS	NS	1.43	hsa-miR-422b	-1.37	NS	-1.47
hsa-miR-331	NS	NS	1.43	hsa-miR-10b	NS	NS	-1.46
hsa-mir-657	NS	NS	1.42	hsa-miR-130a	-1.44	-1.38	-1.45
hsa-miR-487a	NS	1.25	1.42	hsa-mir-597	NS	NS	-1.44
hsa-mir-611	1.48	1.61	1.42	hsa-miR-130b	-1.28	-1.26	-1.43
hsa-mir-596	1.26	1.36	1.4	hsa-mir-580	NS	NS	-1.42
hsa-miR-361	NS	NS	1.4	hsa-miR-33	NS	NS	-1.41
hsa-let-7d	NS	NS	1.38	hsa-miR-526a	-1.88	NS	-1.4
hsa-miR-24	NS	1.42	1.38	hsa-mir-634	-1.36	NS	-1.4
hsa-mir-181d	NS	1.37	1.37	hsa-mir-651	NS	NS	-1.38
hsa-miR-302c*	1.33	1.54	1.36	hsa-mir-765	NS	NS	-1.36
hsa-mir-769	NS	NS	1.36	hsa-miR-100	NS	NS	-1.36
hsa-mir-421	NS	1.28	1.35	hsa-mir-770	NS	NS	-1.34
hsa-miR-500	NS	NS	1.35	hsa-miR-192	NS	NS	-1.33
hsa-let-7g	NS	NS	1.34	hsa-miR-363*	NS	NS	-1.33
hsa-miR-377	NS	NS	1.33	hsa-miR-510	-1.39	-1.39	-1.32
hsa-mir-768	1.31	NS	1.33	hsa-miR-488	-1.18	NS	-1.32
hsa-mir-650	NS	1.29	1.31	hsa-miR-214	NS	NS	-1.31
hsa-miR-485-5p	NS	NS	1.3	hsa-mir-617	NS	NS	-1.3
hsa-mir-801	1.25	NS	1.29	hsa-miR-381	-1.33	-1.32	-1.3
hsa-mir-663	1.43	NS	1.29	hsa-miR-302b*	NS	NS	-1.29
hsa-mir-591	NS	1.24	1.29	hsa-mir-635	NS	-1.37	-1.28
hsa-miR-433	NS	NS	1.28	hsa-miR-518f*	NS	NS	-1.28
hsa-mir-662	NS	1.28	1.28	hsa-mir-632	-2.03	NS	NS
hsa-miR-409-3p	NS	1.08	1.27	hsa-miR-128a	-1.79	NS	NS
hsa-miR-29b	NS	NS	1.26	hsa-mir-640	-1.68	-1.49	NS
hsa-miR-373*	1.33	1.28	1.26	hsa-miR-142-5p	-1.52	NS	-1.26
hsa-miR-378	NS	NS	1.25	hsa-mir-610	-1.4	NS	NS
hsa-mir-411	NS	NS	1.25	hsa-miR-520a*	-1.36	NS	NS
hsa-mir-588	2.36	NS	NS	hsa-miR-133b	-1.34	NS	NS
hsa-mir-578	2.08	NS	NS	hsa-mir-628	-1.3	-1.26	NS
hsa-miR-492	1.34	NS	NS	hsa-miR-9*	-1.29	NS	NS
hsa-miR-221	1.32	NS	NS	hsa-miR-513	-1.27	-1.26	NS
hsa-mir-602	1.24	NS	NS	hsa-miR-185	-1.25	-1.22	NS
hsa-miR-328	1.2	NS	NS	hsa-miR-124a	-1.24	NS	NS
hsa-miR-502	1.2	NS	NS	hsa-miR-125b	-1.23	-1.19	NS
hsa-mir-551b	NS	1.86	NS	hsa-miR-526a	-1.23	NS	NS
hsa-miR-340	NS	1.64	NS	hsa-mir-376b	NS	-1.56	NS
hsa-mir-572	1.35	1.58	NS	hsa-miR-412	NS	-1.44	NS
hsa-miR-200a*	NS	1.47	NS	hsa-miR-10a	NS	-1.38	NS
hsa-mir-614	1.33	1.32	NS	hsa-miR-193b	NS	-1.27	NS
hsa-miR-498	NS	1.32	NS	hsa-miR-453	NS	-1.24	NS
hsa-miR-222	NS	1.28	NS	hsa-mir-584	NS	-1.2	NS
hsa-miR-34b	NS	1.25	NS	hsa-mir-575	NS	-1.2	NS
hsa-miR-27a	NS	1.24	NS	hsa-mir-582	NS	-1.14	NS
hsa-mir-660	NS	1.24	NS				
hsa-mir-675	NS	1.21	NS				
hsa-mir-551a	1.2	1.17	NS				

NS = not significant

**Figure 4:**
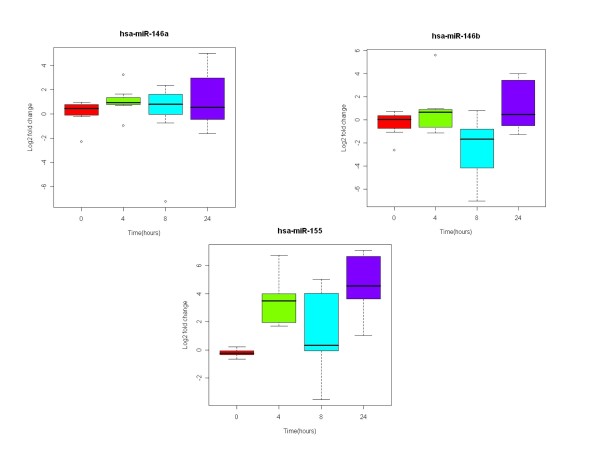
**Change in the expression of miR-146a, -146b, and -155 during DC maturation**. iDCs from 6 healthy subjects sampled after 0, 4, 8 and 24 hours of culture in LPS and IFN-γ were analyzed by quantitive RT-PCR for the expression of miR-146a, -146b, and -155.

To validate the miR microarray results, miR-146a, miR-146b, miR-155, were selected for analysis by quantitative RT-PCR. These miR was selected because they have been previously found to be expressed by macrophages or DCs [19-21]. The results were consistent with those obtained with the microarrays (Figure 4).

### Proteins Produced during DC maturation

**Figure 5:**
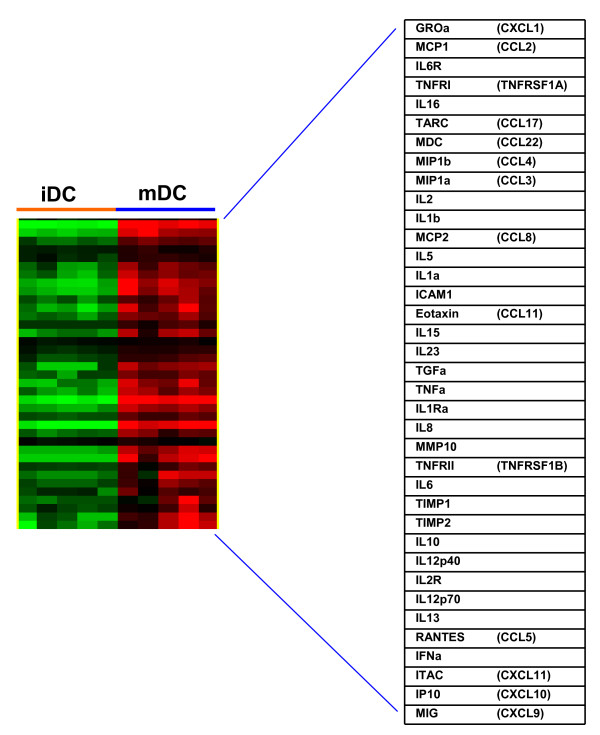
**Cytokine, chemokine and growth factors production by cultured DCs**. The supernants from the 6 healthy subject iDCs and mDCs from 6 healthy subjects were analyzed for 50 cytokines, chemokines and growth factors using an ELISA assay. The levels of 36 soluble factors differed between iDCs and mDCs (t-tests, p < 0.05), the levels of all 36 were greater in mDCs. The differentially expressed factors were analyzed by unsupervised hierachical clustering analysis.

The levels of 50 proteins were measured in DC culture supernatants at time 0 and after 24 hours of maturation. The proteins whose levels changed significantly (t-tests, p < 0.05) were visualized by a heatmap (Figure 5). The levels of 16 proteins related to the DC function increased including CXCL1 (GROα), CCL2 (MCP1), CCL3 (MIP1α), CCL4 (MIP1β), CCL5 (RANTES), CCL8 (MCP2), CCL11 (Eotaxin), CCL17 (TARC), CCL22 (MDC), CXCL9 (MIG), CXCL10 (IP10), CXCL11 (ITAC), IL-6, IL-8, IL-10, IL-12 and TNF-α [see Additional File 4, Table S4]. These results are consistent with the changes in gene expression levels.

### Mature DC function testing and cytokine detection

**Figure 6:**
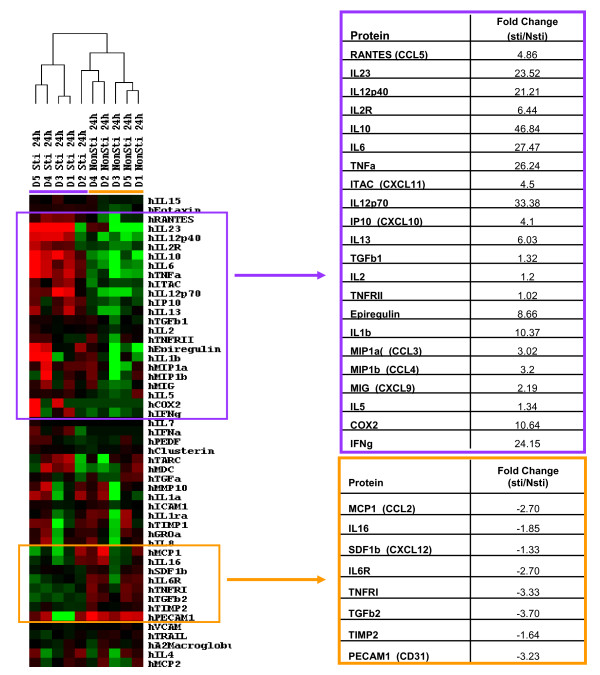
**Production of cytokine, chemokine and growth factors by stimulated mDCs**. Mature DCs from 6 healthy subjects produced by incubation with LPS and IFN-γ were incubated with mouse fibroblasts transfected with CD40-Ligand (CD40L-LTK). The supernant from the stimulated mDCs and unstimulated mDCs were analyzed for 50 cytokines, chemokines and growth factors using an ELISA assay. The levels of the 50 soluble factors were analyzed by unsupervised hierachical clustering analysis. The DCs separated into stimulated mDCs (purple bar) and unstimulated mDCs (orange bar). The cluster of factors that were increased in stimulated mDCs are shown in the purple box and those decreased in stimulated mDCs are shown in the orange box.

To test the function of mature DCs, we incubated mDCs with mouse fibroblasts transfected to express human CD40-Ligand (CD40L-LTK) and compared supernatant factor levels in CD40L-LTK-stimulated mDCs with unstimulated mDCs. The levels of two important cytokines related to DC function, IL-12 and IL-10, increased more than 20-fold post-stimulation (Figure 6). We also observed that the levels of cytokines and chemokines involved in regulating inflammatory and immune responses were elevated. These factors included: IL-1β, IL-2, IL-5, IL-6, IL-13, IL-23, IL-1β, IFN-γ, TNF-α, CCL3 (MIP1α), CCL4 (MIP1β), CCL5 (RANTES), CXCL9 (MIG), CXCL10 (IP10), and CXCL11 (ITAC) [see Additional File 5, Table S5]. These findings are consistent with the results of result of gene expression profiling.

## Discussion

The use of DC-based cellular therapies to enhance innate and adoptive immune mediated tumor rejection is a very promising regimen which has shown evidence of improving patient survival and objectively enhancing tumor elimination. Numerous DC maturation protocols have been developed and each one has unique features to enhance DC function. In this study, we used a classical iDC generation procedure that makes use of GM-CSF plus IL-4 stimulation which was followed by LPS plus IFN-γ maturation. We studied changes in gene and miR expression in maturing DCs to characterize the nature of the mDCs produced with LPS and IFN-γ and to identify genes and miR that could serve as biomarkers for the characterization mDCs

Our study demonstrated that after 24 hours of stimulation with LPS and IFN-γ, mDCs expressed increased levels of HLA Class I and Class II antigens as well as the costimulatory molecules CD80, CD86 and the chemotaxic receptor CCR7. The mDCs were also well-armed to induce Th1 responses as exemplified by significant elevations in the expression of the Th1 cell attractants CXCL9, CXCL10, CXCL11 and CCL5. Another factor used for DC maturation, prostaglandin E2 (PGE2), induces mDCs which produced high levels of the regulator T cell (Treg) attracting cytokines CCL22 and CXCL12 [22]. These Treg cells can counter the effects of Th1 responses by cytotoxic T cells, Th1 cells, and NK cells. In contrast, we found that LPS and IFN-γ maturated DCs did not increase the levels of CCL22 and CXCL12 expression.

We found that the expression of a number of other genes were up-regulated during DC maturation. The up-regulated genes during DC fell into three general categories: those that were up-regulated to a similar level throughout maturation, those that were most up-regulated early in maturation and those that were most up-regulated after 24 hours of maturation. Genes whose expression was up-regulated throughout maturation were most likely to belong to several pathways involved with inflammation: interferon signaling, IL-10 signaling, CD40 signaling, IL-6 signaling, activation of IRF by cytosolic pattern recognition receptors and role of pattern recognition receptors in recognition of bacteria and virus pathways. Specific genes that were up-regulated throughout maturation include CCL3, CCL4, CCL5, CCL8, CXCL10, CXCL11, CCR7, IL-1b, IL-6, IL-15, IL-27, IL-7R, IL-10RA, IL-15RA, STAT1, STAT2, STAT3, CD80, CD83, and CD86. Among the genes that were markedly up-regulated (more than 10-fold) during maturation and are good potential mDC biomarkers are CCL5, CXCL10, CCR7, IFI44L, IFIH1, MX1, ISG15, ISG20, INDO, MT2A, TRAF1, BRIC3, USP18, and CD83 (Table 3). CCL5, CCR7, and CD83 may be particularly good potency biomarker candidates because they have important roles in DC function.

**Table 3:** Genes up-regulated during DC maturation that could be used as biomarkers for assessing mDCs

	Fold increase in gene expression for each maturation time*						
**Gene**	**4 hours**	**8 hours**	**24 hours**	**Gene**	**4 hours**	**8 hours**	**24 hours**

**Up-regulated to a similar degree throughout maturation**				**Up-regulated most early in DC maturation**			

aCCL5	108	148	94.1	IL6	13.1	11.3	5.15
CXCL10	28.5	31.8	21.2	IL8	78.0	70.3	12.7
CCR7	10.5	11.5	18.2	IL7R	29.1	29.9	10.5
IL15	7.12	5.54	8.13	CCL4	92.3	53.4	6.91
IFI27	6.99	7.62	10.2	TNFAIP6	30.0	18.7	10.7
IFI44L	14.8	16.7	20.5	IFIT3	36.2	22.5	10.5
IFIH1	16.9	9.42	11.3	OASL	68.1	44.1	30.7
IFIT1	29.8	27.0	21.7	GBP1	66.2	35.3	30.2
MX1	18.4	15.6	14.1	HES4	229	115	37.4
ISG15	50.6	58.3	41.8	**Up-regulated most late in DC maturation**			
				
ISG20	94.1	87.9	62.9	CCL8	11.3	31.8	31.2
IRF7	9.77	9.38	12.0	EBI3	17.6	21.8	34.6
GBP4	36.3	21.2	20.2	IFITM1	13.2	22.5	48.6
DUSP5	21.7	15.8	22.6	MT1B	10.2	10.5	20.6
NFKBIA	11.7	13.3	10.4	MT1E	NS	1.78	46.1
ATF3	10.2	5.38	11.4	MT1G	NS	2.77	42.3
TNFSF10	19.4	14.8	13.8	MT1H	22.7	20.5	62.6
TNFRSF9	8.13	6.39	10.2	GADD45A	NS	11.6	50.7
SOD2	51.6	58.4	28.0	CD200	2.49	4.94	15.1
CD38	8.35	9.26	9.02	LAMP3	11.7	17.5	37.4
CD44	3.48	1.76	2.18	RGS1	5.70	18.1	28.3
CD80	3.14	2.93	3.49	SAT1	3.79	6.26	18.1
CD83	22.0	17.3	23.6	CYP27B1	6.59	12.5	21.4
CD86	1.62	1.32	2.34	RIPK2	10.7	14.0	23.1
				
INDO	28.6	18.6	16.3				
MT2A	54.3	72.0	69.2				
TRAF1	31.4	16.7	24.4				
GADD45B	17.8	10.7	10.2				
MT1M	8.26	8.40	15.1				
MT1P2	14.6	13.9	20.3				
BIRC3	23.0	18.4	28.2				
USP18	34.7	29.9	29.8				
TUBB2A	10.7	8.19	10.4				

Fold-increase compared to iDCs.

Genes whose expression was most up-regulated early in maturation included genes in the NF-kB signaling; IL-6, IL-8, IL-10, IL-15 and IL-17 signaling; 4-1 bb signaling in T lymphocytes; MIF regulation of innate immunity; and role of pattern recognition receptors in the recognition of bacteria and viruses pathways. Specific genes that were most up-regulated early in maturation include CXCL1, IL-1α, TNF, TNFSF8, TNFAIP5, TNIP3, TRAF3, JAK2, BID, CASP1, LILRB1, LILRB2, IILRB3, 2NF422, MMP-10, IL-10, and IL-12b. Genes whose expression was markedly up-regulated early and are good biomarker candidates include: CCL4 (MIP-1b), HES4, GBP1, OSAL, IFIT3, IL-8, IL7R, and TNFAIP6 (Table 3).

The DC genes that were most up-regulated after 24-hours of stimulation, in general, included genes that belonged to metabolic pathways. However, a number of inflammatory pathway genes were also in this group. Genes in this group included CXCR4, IFITM4P, IFITM1, GADD45A, LAMP3, TRAF5, STAT5, CASP3, GZMB, MTIB, MTIE, MTIG, MTIH, CCL8, HLA-A, HLA-B, HLA-C, and LYGE. Among these genes GADD45A, MTIE, and MTIG were not up-regulated after 4 hours, but were markedly up-regulated after 24 hours and may be especially good biomarker candidates (Table 3).

Some genes were markedly down-regulated in mDCs including CD1C, MAF, and CLEC10A (Table 4). These genes are also mDC biomarker candidates. The expression of MHC Class II genes was down-regulated during maturation, but flow cytometer analysis showed that the cell surface expression of HLA-DR protein increased during maturation (Table 1). This observation suggests an active regulation of these genes at the transcription level. These transcripts could be sensored by the encoded protein and regulatory miR. This observation could also be explained by the fact that the majority of MHC II molecules are stored intracellularly within the internal vesicles of multivesicular bodies in iDCs. Thus MHC II antigen expression can increase while gene expression decreases.

**Table 4:** Genes down-regulated during DC maturation that could be used as biomarkers for assessing mDCs

	Fold decrease in gene expression for each maturation time*		
	
Gene	4 hours	8 hours	24 hours
**Genes down-regulated to a similar degree throughout maturation**			
CD1C	64.0	82.2	57.8
MYC	9.17	8.83	9.35
MAF	15.0	8.30	20.5
PTGS1	5.43	20.4	13.0
DOK2	8.78	7.03	9.63

**Genes down-regulated most late in DC maturation**			
TGFBI	2.44	6.76	11.2
GATM	2.70	12.0	17.7
ARHGDIB	2.37	10.1	15.4
MRC1	NS	7.15	30.22
CLEC10A	3.82	28.2	43.5

Fold-increase compared to iDCs.

Many cellular therapy laboratories use the production of IL-10 and IL-12 as mDC potency assays. We also found that the mDCs produced soluble IL-10 and IL-12. However, the expression of the genes encoding IL-10 and IL-12B(p40) were up-regulated 3- to 6-fold after 4 and 8 hours of LPS and IFN-γ stimulation, but returned to baseline levels after 24 hours suggesting that these genes may not be good molecular biomarkers.

miRs are endogenously encoded regulatory RNA which regulate mRNA by targeting their 3'UTR and inducing mRNA degradation or protein translation suppression. They are highly involved in development timing, differentiation, and cell cycle regulation. To understand how miR expression is involved in DC maturation, we used miR array analysis. Unlike gene expression analysis, miR expression analysis of maturing DCs revealed two distinct patterns: down-regulation of groups of miR at 8 hours of maturation with sustained low expression throughout the rest of maturation and up-regulation of other groups of miR at 8 hours of maturation and sustained up-regulation. Among the up-regulated miR, the best candidate for potency testing is miR-155. The expression of miR-155 increased more than any other miR with 3-fold up-regulation after 4 hours, 4-fold after 8 hours and 8-fold after 24 hours. This finding is supported by previous reports that miR-155 expression is increased in DC maturation [19-21]. Other miRs that may be good biomarkers are miR-146a and miR-146b, which we also found were up-regulated during DC maturation. These two miRs have also been found to be up-regulated in DCs matured with IL-1β, IL-6, TNFα and PGE2 [21].

Since miR control the expression of multiple genes and proteins, they may actually be better biomarkers of potency then single genes or proteins. miR-155 is located within the noncoding B cell integration cluster (Bic) gene [23] and is functionally important in B cell, T cell and macrophage biology. miR-155 is up-regulated in B cells exposed to antigen, in T cells stimulated by Toll-like receptor ligand and in macrophages by IFN-γ stimulation[24,25]. The Toll-like receptor/interleukin-1 (TRL/IL-1) inflammatory pathway appears to be a general target of miR-155 [19]. One of the genes that it directly targets is the DC transcription factor PU.1 [20]. Furthermore, miR-155 directly controls TAB2 a signal transduction molecule. miR-155 may be part of a negative feedback loop which down modules inflammatory cytokine production including IL-1β in response to LPS-stimulation [19]. Hence, miR-155 may be a particularly good mDC potency biomarker.

The ability of these studies to identify mDC biomarkers is some what limited by the variability of methods used to produce iDCs and mDCs among various laboratories. We used a 3 day DC differentiation protocol that uses IL-4 and GM-CSF followed by differentiation with LPS and IFN-γ. This method is very similar to that used in several clinical vaccine protocols involving mature DCs. However, other protocols use 5 to 8 days of IL-4 and GM-CSF culture to produce iDCs and a variety of other agents are being used for DC maturation. We also used FCS rather than human AB serum in these studies and this could have had some effects on DC maturation.

In conclusion, we found that LPS and IFN-γ induced mDCs expressed large quantities of Th1 attractants, but not Treg attractants, suggesting that these mDCs will be particularly effective for adoptive immune cancer therapy. In addition, we identified several genes and miRs that may be useful biomarkers for consistency, comparability, and potency testing. However, further studies are needed to validate their utility as biomarkers.

## Competing interests

The authors declare that they have no competing interests.

## Authors' contributions

PJ participated in the design of the study, performed experiments, analyzed the data and participated in writing the manuscript. THH participated in the design of the study, performed experiments, analyzed the data and participated in writing the manuscript. JR particapted in designing the study, performed experiments and analyzed data. SS performed experiments and analyzed data. EW participated in designing the study and the writing of the manuscript. FMM participated in designing the study and the writing of the manuscript. DFS participated in designing the study, coordinating the study and the writing of the manuscript. All authors have read and approved the final manuscript.

## Supplementary Material

Additional file 1 **Table S1: The 30 canonical pathways with the most differentially expressed DC genes for each of the 5 gene clusters**. Canonical pathway analysis showed that genes in each of these 5 clusters belong to different pathways. Click here for file

Additional file 2 **Table S2. Immature DC Genes whose expression was up-regulated following LPS and IFN-γ stimulation**. The specific genes that were differentially expressed among the DCs stimulated with LPS and IFN- γ for different durations of time and their fold-change, up-regulated genes summary. (t-test, p ≤ 0.001 compared to hr 0). Click here for file

Additional file 3 **Table S3. Immature DC Genes whose expression was down-regulated following LPS and IFN- γ stimulation**. The specific genes that were differentially expressed among the DCs stimulated with LPS and IFN- γ for different durations of time and their fold-change, down-regulated genes summary. (t-test, p ≤ 0.001 compared to hr 0). Click here for file

Additional file 4 **Table S4. Soluble factor levels in DC cell culture supernatant whose expression was up-regulated following LPS and IFN- γ stimulation**. Soluble factor levels in DC cell culture supernatant whose expression was up-regulated following LPS and IFN- γ stimulation. Click here for file

Additional file 5 **Table S5. Soluble factor levels and fold changes in mature DC culture supernatant after 24 hours of CD40 Ligand stimulation**. Soluble factor levels and fold changes in mature DC culture supernatant after 24 hours of CD40 Ligand stimulation. Click here for file
