# Supplementary material for: Molecular signatures of maturing dendritic cells: implications for testing the quality of dendritic cell therapies
Source: J Transl Med. 2010 Jan 15;8:4. doi: 10.1186/1479-5876-8-4 (PMC2841589; doi:10.1186/1479-5876-8-4)
Supplement: Additional file 1 — Table S1: The 30 canonical pathways with the most differentially expressed DC genes for each of the 5 gene clusters. Canonical pathway analysis showed that genes in each of these 5 clusters belong to different pathways. [file 1479-5876-8-4-S1.DOC]

**Table S1: The 30 canonical pathways with the most differentially expressed DC genes for each of the five gene clusters**

|  | **Cluster 1** | **Cluster 2** | **Cluster 3** | **Cluster 4** | **Cluster 5** |
| --- | --- | --- | --- | --- | --- |
| **1** | Glucocorticoid Receptor Signaling | **Activation of IRF by Cytosolic Pattern Recognition Receptors*** | **Arginine and Proline Metabolism†** | **Alanine and Aspartate Metabolism†** | **Oxidative Phosphorylation†** |
| **2** | **NF-κB Signaling*** | **Interferon Signaling*** | **One Carbon Pool by Folate†** | **Pyrimidine Metabolism†** | **Fatty Acid Elongation in Mitochondria†** |
| **3** | **IL-17 Signaling*** | **Role of Pattern Recognition Receptors in Recognition of Bacteria and Viruses*** | **Pyrimidine Metabolism†** | **Aminoacyl-tRNA Biosynthesis†** | Mitochondrial Dysfunction |
| **4** | **MIF Regulation of Innate Immunity*** | **TREM1 Signaling*** | Synaptic Long Term Potentiation | Nucleotide Excision Repair Pathway | *Antigen Presentation Pathway$* |
| **5** | **IL-8 Signaling*** | Death Receptor Signaling | **Inositol Metabolism†** | **Biosynthesis of Steroids†** | Leukocyte Extravasation Signaling |
| **6** | Nicotinate and Nicotinamide Metabolism | **IL-10 Signaling*** | **Alanine and Aspartate Metabolism†** | Interferon Signaling | Clathrin-mediated Endocytosis |
| **7** | **Toll-like Receptor Signaling*** | Protein Ubiquitination Pathway | **β-alanine Metabolism†** | CD27 Signaling in Lymphocytes | **Purine Metabolism†** |
| **8** | **IL-6 Signaling*** | Role of RIG1-like Receptors in Antiviral Innate Immunity | 14-3-3-mediated Signaling | **N-Glycan Degradation†** | Eicosanoid Signaling |
| **9** | B Cell Receptor Signaling | Induction of Apoptosis by HIV1 | **Butanoate Metabolism†** | Estrogen Receptor Signaling | CTLA4 Signaling in Cytotoxic T Lymphocytes |
| **10** | **IL-10 Signaling*** | **CD40 Signaling*** | **Glutamate Metabolism†** | α -Adrenergic Signaling | **Xenobiotic Metabolism Signaling†** |
| **11** | **IL-15 Production*** | **Role of PKR in Interferon Induction and Antiviral Response*** | **Nitrogen Metabolism†** | **Glycerolipid Metabolism†** | CD28 Signaling in T Helper Cells |
| **12** | PI3K/AKT Signaling | Glucocorticoid Receptor Signaling | **Pyruvate Metabolism†** | **Glycolysis/ Gluconeogenesis†** | *IL-4 Signaling$* |
| **13** | *Acute Phase Response Signaling#* | B Cell Activating Factor Signaling | **Lysine Degradation†** | **Glycosaminoglycan Degradation†** | α-Adrenergic Signaling |
| **14** | **4-1BB Signaling in T Lymphocytes*** | Hepatic Fibrosis / Hepatic Stellate Cell Activation | Fcγ Receptor-mediated Phagocytosis in Macrophages and Monocytes | IL-22 Signaling | **Glutathione Metabolism†** |
| **15** | **Role of Pattern Recognition Receptors in Recognition of Bacteria and Viruses*** | *Acute Phase Response Signaling#* | **Inositol Phosphate Metabolism†** | Pentose Phosphate Pathway | **Methane Metabolism†** |
| **16** | Lysine Biosynthesis | **IL-6 Signaling*** | Fc Epsilon RI Signaling | Synthesis and Degradation **of Ketone Bodies†** | Integrin Signaling |
| **17** | Apoptosis Signaling | **IL-9 Signaling*** | Erythropoietin Signaling | **Purine Metabolism†** | *LPS/IL-1 Mediated Inhibition of RXR Function$* |
| **18** | **Role of PKR in Interferon Induction and Antiviral Response*** | JAK/Stat Signaling | Role of NFAT in Regulation of the Immune Response | TGF-β Signaling | *Role of NFAT in Regulation of the Immune Response$* |
| **19** | PPAR Signaling | **4-1BB Signaling in T Lymphocytes*** | Huntington's Disease Signaling | **Arginine and Proline Metabolism†** | Ephrin Receptor Signaling |
| **20** | N-Glycan Biosynthesis | Lymphotoxin β Receptor Signaling | **Ascorbate and Aldarate Metabolism†** | NRF2-mediated Oxidative Stress Response | **Arachidonic Acid Metabolism†** |
| **21** | **p38 MAPK Signaling*** | **IL-15 Production*** | Chemokine Signaling | PI3K/AKT Signaling | **Valine, Leucine and Isoleucine Degradation†** |
| **22** | Wnt/β-catenin Signaling | **NF-κB Signaling*** | Protein Ubiquitination Pathway | **Fructose and Mannose Metabolism†** | T Helper Cell Differentiation |
| **23** | *Airway Pathology in Chronic Obstructive Pulmonary Disease#* | VDR/RXR Activation | **Methionine Metabolism†** | **Galactose Metabolism†** | CCR5 Signaling in Macrophages |
| **24** | Axonal Guidance Signaling | Amyotrophic Lateral Sclerosis Signaling | **Purine Metabolism†** | **Butanoate Metabolism†** | Macropinocytosis |
| **25** | **Role of NFAT in Regulation of the Immune Response*** | Apoptosis Signaling | B Cell Receptor Signaling | **Propanoate Metabolism†** | Fcγ Receptor-mediated Phagocytosis in Macrophages and Monocytes |
| **26** | Ephrin Receptor Signaling | Hepatic Cholestasis | CTLA4 Signaling in Cytotoxic T Lymphocytes | CXCR4 Signaling | **Cysteine Metabolism†** |
| **27** | **IL-12 Signaling and Production in Macrophages*** | PPAR Signaling | **D-glutamine and D-glutamate Metabolism†** | **Glycosphingolipid Biosynthesis - Ganglioseries†** | **Sulfur Metabolism†** |
| **28** | ERK/MAPK Signaling | *IL-3 Signaling#* | VEGF Signaling | **Methionine Metabolism†** | **N-Glycan Biosynthesis†** |
| **29** | Lymphotoxin β Receptor Signaling | **IL-17 Signaling*** | IL-2 Signaling | **Lysine Degradation†** | **Fatty Acid Metabolism†** |
| **30** | **TREM1 Signaling*** | *Airway Pathology in Chronic Obstructive Pulmonary Disease#* | **Propanoate Metabolism†** | B Cell Activating Factor Signaling | **Glycosphingolipid Biosynthesis - Globoseries†** |

*= cellular immune response pathways † = metabolism pathways

# = cytokine signaling pathways $ = Humoral immune response and pathogen-influenced signaling pathway
