# Supplementary material for: Molecular signatures of maturing dendritic cells: implications for testing the quality of dendritic cell therapies
Source: J Transl Med. 2010 Jan 15;8:4. doi: 10.1186/1479-5876-8-4 (PMC2841589; doi:10.1186/1479-5876-8-4)
Supplement: Additional file 2 — Table S2. Immature DC Genes whose expression was up-regulated following LPS and IFN-γ stimulation. The specific genes that were differentially expressed among the DCs stimulated with LPS and IFN- γ for different durations of time and their fold-change, up-regulated genes summary. (t-test, p ≤ 0.001 compared to hr 0). [file 1479-5876-8-4-S2.DOC]

**Table S2. Immature DC Genes whose expression was regulated following LPS and IFN-γ stimulation**

|  | **Gene** | **Gene expression fold change** | | |  | **Gene** | **Gene expression fold change** | | |  | **Gene** | **Gene expression fold change** | | |
| --- | --- | --- | --- | --- | --- | --- | --- | --- | --- | --- | --- | --- | --- | --- |
| **Category** | **Symbol** | **4 h** | **8 h** | **24h** | **Category** | **symbol** | **4h** | **8h** | **24h** | **Category** | **symbol** | **4 h** | **8 h** | **24 h** |
| Chemokine | CCL2 | 2.34 | 1.97 | NS | Interferon | IFI4 | 2.47 | 2.39 | 2.58 | MAPK | GADD45A | NS | 11.6 | 50.7 |
| Ligands & | CCL3 | 27.6 | 12.0 | 3.76 | Induced or | IFI27 | 6.99 | 7.62 | 10.24 | Signaling | GADD45B | 17.8 | 10.7 | 10.2 |
| Receptors | CCL4 | 92.3 | 53.4 | 6.91 | Stimulated | IFI35 | 1.63 | N | N | Pathway | DUSP1 | 11.6 | 4.98 | NS |
|  | CCL4L1 | 32.7 | 20.5 | 3.95 | Proteins | IFI41 | 4.27 | 2.91 | 2.61 | Related | DUSP2 | 15.2 | NS | NS |
|  | CCL5 | 108 | 148 | 94.1 |  | IFI44 | 6.04 | 3.58 | 3.79 |  | DUSP4 | 2.04 | NS | NS |
|  | CCL8 | 11.3 | 31.8 | 31.2 |  | IFI44L | 14.8 | 16.7 | 20.5 |  | DUSP5 | 21.7 | 15.8 | 22.6 |
|  | CCL17 | NS | NS | 1.79 |  | IFIH1 | 16.9 | 9.42 | 11.3 |  | RAC1 | 1.89 | 2.02 | 2.25 |
|  | CXCL1 | 33.5 | 25.5 | NS |  | IFIT1 | 29.8 | 27.0 | 21.7 |  | CDC42 | 1.62 | NS | NS |
|  | CXCL2 | 5.24 | 5.54 | 2.08 |  | IFIT2 | 7.40 | 4.44 | 3.42 |  | CDC42EP | 2.40 | 5.13 | 2.74 |
|  | CXCL9 | 25.7 | 9.28 | N S |  | IFIT3 | 36.2 | 22.5 | 10.5 |  | CDGAP | 1.75 | NS | NS |
|  | CXCL10 | 28.5 | 31.8 | 21.21 |  | IFIT4 | 2.47 | 2.39 | 2.58 |  | ARHGAP17 | 1.48 | NS | NS |
|  | CXCL11 | 37.1 | 13.4 | 3.24 |  | IFIT5 | 4.47 | 2.64 | 2.38 |  | MAP2K1IP1 | NS | NS | 2.56 |
|  | CCR7 | 10.5 | 11.5 | 18.2 |  | IFITM1 | 13.2 | 22.5 | 48.6 |  | MAP3K8 | 4.42 | NS | NS |
|  | CXCR4 | NS | NS | 3.04 |  | IFITM2 | 4.35 | 6.45 | 7.86 |  | MAP2K6 | 1.52 | NS | NS |
|  |  |  |  |  |  | IFITM4P | NS | 1.63 | 1.53 |  | STK4 | 2.30 | 2.38 | 3.11 |
| Cytokines | IL1A | 3.41 | NS | NS |  | MX1 | 18.4 | 15.6 | 14.1 |  |  |  |  |  |
| & receptors | IL1B | 19.7 | 11.9 | 4.32 |  | ISG15 | 50.6 | 58.3 | 41.8 | NF-Kb | IKBKA | 1.83 | NS | NS |
|  | IL6 | 13.1 | 11.3 | 5.15 |  | ISG20 | 94.1 | 87.9 | 62.9 |  | NFKBIA | 11.7 | 13.3 | 10.4 |
|  | IL8 | 78.0 | 70.3 | 12.7 |  | OAS1 | 6.01 | 5.62 | 6.79 |  | NFKBIB | 2.29 | 1.87 | 1.95 |
|  | IL10 | 4.81 | 6.35 | NS |  | OAS3 | 11.6 | 9.88 | 7.26 |  | RELB | 3.53 | 3.69 | 7.09 |
|  | IL12B | 3.51 | 3.60 | NS |  | OASL | 68.1 | 44.1 | 30.7 |  | NFKB1 | 15.56 | 7.08 | 6.30 |
|  | IL15 | 7.12 | 5.54 | 8.13 |  |  |  |  |  |  | NFKB2 | 2.86 | 2.39 | NS |
|  | IL27 | 19.5 | 6.93 | 4.03 | Interferon | IRF1 | 7.75 | 7.04 | 8.89 |  |  |  |  |  |
|  | IL7R | 29.1 | 29.9 | 10.5 | regulator | IRF2 | 1.71 | 1.81 | NS | Activation | ATF3 | 10.2 | 5.38 | 11.4 |
|  | IL10RA | 3.58 | 1.83 | 2.38 | Factors | IRF4 | NS | NS | 4.29 | Protein-1 | ATF5 | 2.73 | NS | NS |
|  | IL15RA | 7.59 | 6.56 | 8.25 |  | IRF7 | 9.77 | 9.38 | 12.0 |  | BATF | 16.41 | 9.31 | 2.34 |
|  | IL21R | 2.29 | NS | NS |  | IRF8 | 5.78 | NS | NS |  | JDP1 | 2.26 | 1.66 | 2.67 |
|  | IL22RA1 | 3.35 | 1.78 | NS |  | IRF9 | 2.09 | 2.15 | 2.77 |  | JDP2 | 2.53 | NS | NS |
|  | CRLF2 | 4.81 | 4.88 | 5.73 |  |  |  |  |  |  | MAFF | NS | NS | 2.26 |
|  | EBI3 | 17.6 | 21.8 | 34.6 | Guanylate | GBP1 | 66.2 | 35.3 | 30.2 |  |  |  |  |  |
|  |  |  |  |  | Binding | GBP2 | 4.80 | 2.79 | 2.43 | Apoptosis | BAG1 | 1.87 | NS | NS |
| TNF | TNF | 37.4 | 8.78 | NS | Protein | GBP3 | 13.48 | 7.25 | 6.43 | Related | BCL2A1 | 22.79 | 6.27 | 1.95 |
| Superfamily | TNFSF8 | 5.06 | NS | NS |  | GBP4 | 36.3 | 21.2 | 20.2 |  | MCL1 | 5.24 | 4.84 | 4.64 |
| & TNFR | TNFSF9 | 2.76 | NS | NS |  | GBP5 | 57.17 | 11.4 | 8.28 |  | BCL2L7 | 1.43 | NS | NS |
| Superfamily | TNFSF10 | 19.4 | 14.8 | 13.8 |  |  |  |  |  |  | BCL2L14 | NS | 1.42 | 1.77 |
|  | TNFSF13B | 9.93 | 4.24 | 2.86 | TLR | TICAM1 | 4.61 | 2.59 | 2.28 |  | BID | 8.96 | 3.84 | NS |
|  | TNFRSF1B | 2.11 | NS | NS | Signaling | TICAM2 | 3.41 | 2.48 | NS |  | BIRC2 | 3.22 | 3.08 | 3.12 |
|  | TNFRSF4 | 12.9 | 6.65 | 3.12 |  | IRAK3 | NS | 2.52 | NS |  | BIRC3 | 23.0 | 18.4 | 28.2 |
|  | TNFRSF5 | 6.49 | 2.45 | 2.77 |  | IRAK4 | 2.11 | NS | NS |  | CFLAR | 15.3 | 10.3 | 8.44 |
|  | FAS | 1.72 | 2.14 | 2.20 |  | MAP3K7IP2 | 1.55 | NS | NS |  | CASP1 | 2.80 | 2.54 | NS |
|  | TNFRSF6B | 3.45 | 7.43 | 4.43 |  | RIPK2 | 10.7 | 14.0 | 23.1 |  | CASP3 | NS | NS | 2.03 |
|  | TNFRSF9 | 8.13 | 6.39 | 10.2 |  | IKBKE | 1.83 | NS | NS |  | TRADD | 1.94 | 2.81 | 3.91 |
|  | TNFRSE18 | 2.09 | NS | NS |  |  |  |  |  |  | PRF1 | 2.64 | 2.33 | 2.70 |
|  |  |  |  |  | JAK-STAT | JAK1 | NS | NS | 1.81 |  | GZMB | NS | 1.84 | 1.82 |
| TNFα | TNFAIP3 | 17.7 | 11.4 | 8.78 | Pathway | JAK2 | 3.75 | NS | NS |  |  |  |  |  |
| Related | TNFAIP5 | 20.0 | 13.3 | NS |  | STAT1 | 3.24 | 3.91 | 5.02 | Metallo- | MT1A | 11.0 | 7.62 | 7.96 |
| Proteins | TNFAIP6 | 30.0 | 18.7 | 10.7 |  | STAT2 | 5.20 | 5.05 | 2.86 | thioneins | MT1B | 10.2 | 10.5 | 20.6 |
|  | TNFAIP8 | 2.69 | NS | NS |  | STAT3 | 2.35 | 2.72 | 2.47 |  | MT1E | NS | 1.78 | 46.1 |
|  | TNIP1 | NS | NS | 1.90 |  | STAT5A | 4.42 | 2.64 | 3.00 |  | MT1G | NS | 2.77 | 42.3 |
|  | TNIP3 | 13.6 | 4.74 | NS |  | STAT5B | NS | NS | 2.52 |  | MT1H | 22.7 | 20.5 | 62.6 |
|  | LITAF | 4.11 | 3.25 | 2.03 |  | SMARCA4 | 2.72 | NS | NS |  | MT1M | 8.26 | 8.40 | 15.1 |
|  |  |  |  |  |  | NMI | 3.52 | 2.80 | 2.75 |  | MT1P2 | 14.6 | 13.9 | 20.3 |
| TNF | TRAF1 | 31.4 | 16.7 | 24.4 |  | SOCS1 | 1.61 | NS | NS |  | MT2A | 54.3 | 72.0 | 69.2 |
| Receptor | TRAF3 | 31.4 | NS | NS |  | SOCS2 | 4.97 | 4.85 | 5.71 |  |  |  |  |  |
| Associated | TRAF5 | NS | NS | 2.92 |  |  |  |  |  |  |  |  |  |  |
| Factors & | TRAF6 | NS | 1.57 | 2.15 |  |  |  |  |  |  |  |  |  |  |
| Interacting | TRAF3IP2 | NS | 1.57 | NS |  |  |  |  |  |  |  |  |  |  |
| Proteins | TRAF3IP3 | 1.83 | NS | NS |  |  |  |  |  |  |  |  |  |  |
|  |  |  |  |  |  |  |  |  |  |  |  |  |  |  |
| CD | CD38 | 8.35 | 9.26 | 9.02 | Vesicular | STX11 | 12.1 | 6.62 | 4.69 | Aminoacid | CKB | 11.9 | 14.2 | 8.85 |
| Markers | CD44 | 3.48 | 1.76 | 2.18 | Transport | STX17 | 2.10 | 2.21 | 1.90 | metabolism | SAT1 | 3.79 | 6.26 | 18.1 |
|  | CD80 | 3.14 | 2.93 | 3.49 |  | TFRC | 6.60 | 6.05 | 3.45 |  | LAP3 | 2.57 | 2.49 | 2.34 |
|  | CD83 | 22.0 | 17.3 | 23.6 |  |  |  |  |  |  | MAOA | 2.44 | 3.10 | 3.88 |
|  | CD86 | 1.62 | 1.32 | 2.34 | Cell cycle | G0S2 | 17.7 | 10.5 | 3.90 |  | ADC | NS | 1.40 | 2.31 |
|  | CD200 | 2.49 | 4.94 | 15.06 | & Cell - | GADD45A | NS | 11.7 | 50.7 |  | ALAS1 | 3.32 | 3.60 | 4.47 |
|  |  |  |  |  | Signaling | GADD45B | 17.8 | 10.7 | 10.2 |  | PHGDH | NS | NS | 2.76 |
| Antigen | ICAM1 | 4.30 | 5.21 | 4.87 |  | SMCIA | 16.1 | 10.4 | 8.68 |  |  |  |  |  |
| Uptake & | LAMP3 | 11.7 | 17.5 | 37.4 |  | LY6E | NS | 2.83 | 3.94 | Lipid | CYP27B1 | 6.59 | 12.5 | 21.4 |
| Presentation | LILRB4 | 4.48 | 4.33 | 4.01 |  | PTPN1 | 3.08 | 2.69 | 4.63 | metabolism | SPHK1 | 4.04 | 4.96 | 5.59 |
|  | MARCKS | 2.09 | 3.49 | 2.57 |  | PTPN2 | 3.88 | 4.13 | 1.98 |  | LIPA | 5.46 | 5.35 | 4.18 |
|  | MARCKSL1 | 6.58 | 14.2 | 21.6 |  | CDKN1A | 5.39 | 3.61 | 5.77 |  | MGLL | 2.35 | 2.27 | 3.13 |
|  | PNRC1 | 5.07 | 8.14 | 10.2 |  | CDKN2D | NS | 1.52 | 1.70 |  | CEL | NS | NS | 2.69 |
|  | B2M | 2.08 | 2.34 | 4.06 |  | YWHAQ | NS | NS | 3.25 |  |  |  |  |  |
|  | HLA-A | NS | NS | 2.01 |  |  |  |  |  | Zinc finger | ZNFX1 | 6.75 | 4.69 | 5.12 |
|  | HLA-B | NS | NS | 2.63 | G–protein | RGS1 | 5.70 | 18.1 | 28.3 | protein | ZNF258 | NS | NS | 2.92 |
|  | HLA-C | NS | 1.69 | 2.50 | Signaling | RGS16 | 1.82 | 1.99 | NS |  | ZNF364 | 3.53 | 2.34 | 3.21 |
|  | HLA-F | 2.00 | 2.42 | 3.54 |  | RGS20 | 2.34 | NS | NS |  | ZNF423 | 4.09 | 1.81 | NS |
|  | HLA-G | 1.58 | 1.78 | 3.32 |  |  |  |  |  |  | ZNF618 | 2.60 | 2.55 | 3.41 |
|  | HLA-H | 1.96 | 2.15 | 4.03 | Regulation | HES4 | 229 | 115 | 37.4 |  |  |  |  |  |
|  | TAP1 | 5.93 | 3.51 | 4.61 | transcription | BATF | 16.4 | 9.31 | 2.34 | Matrix | MMP10 | 7.16 | 2.46 | NS |
|  | TAP2 | 3.27 | 2.78 | 3.37 |  |  |  |  |  | Metallo- | MMP19 | 4.71 | 2.95 | 2.02 |
|  | PSME2 | 4.19 | 5.61 | 5.49 | Growth | GFI1 | NS | NS | 2.22 | peptidase |  |  |  |  |
|  |  |  |  |  | factors & | LTBP4 | 1.36 | 1.43 | 2.14 |  |  |  |  |  |
| c-type lectin | CLEC2D | 1.54 | 1.60 | 2.38 | Receptors | PDGFRL | 2.07 | 2.03 | 2.53 | Others | USP18 | 34.7 | 29.9 | 29.8 |
| Superfamily | CLEC16A | NS | NS | 1.93 |  |  |  |  |  |  | GCH1 | 21.6 | 11.4 | 5.53 |
|  |  |  |  |  | Oncogene | RAB7A | 3.30 | 5.58 | 7.49 |  | TUBB2A | 10.7 | 8.19 | 10.4 |
| ILR | LILRB1 | 7.44 | 5.42 | NS |  | RAB7L1 | 4.45 | 4.32 | 3.83 |  |  |  |  |  |
| Family | LILRB2 | 13.5 | 9.07 | NS |  | RAP2C | 2.11 | 3.08 | 2.03 |  |  |  |  |  |
|  | LILRB3 | 3.16 | 2.41 | NS |  | PIM1 | 3.09 | 2.22 | 2.73 |  |  |  |  |  |
|  | LILRB4 | 4.48 | 4.33 | 4.01 |  | JUN | NS | NS | 2.04 |  |  |  |  |  |
|  | LILRA3 | 4.76 | 4.52 | 5.00 |  |  |  |  |  |  |  |  |  |  |
|  | LILRA5 | 8.38 | 6.62 | 1.66 | Purine & | ADA | 14.2 | 8.22 | 2.58 |  |  |  |  |  |
|  |  |  |  |  | Pyrimidine | PNPT1 | 8.86 | 7.55 | 5.53 |  |  |  |  |  |
| Immune | SCO2 | 2.26 | 1.78 | 3.56 | Metabolism | NT5C3 | 3.49 | 3.73 | 5.21 |  |  |  |  |  |
| Related | SOD2 | 51.6 | 58.4 | 28.0 |  | GMPR | 2.53 | 3.59 | 5.56 |  |  |  |  |  |
|  | PTX3 | 20.0 | 13.3 | NS |  |  |  |  |  |  |  |  |  |  |
|  | NCF1 | 8.52 | 6.27 | 3.04 | Tryptophan | WARS | 46.7 | 21.4 | 7.51 |  |  |  |  |  |
|  | OSM | NS | 6.11 | 5.05 | Metabolism | INDO | 28.6 | 18.6 | 16.3 |  |  |  |  |  |
|  | TXN | 3.76 | 5.20 | 4.23 |  | KYNU | 10.9 | 8.31 | 4.19 |  |  |  |  |  |
|  | PTGS2 | 15.6 | 5.16 | 2.41 |  | SIAH2 | 2.19 | 2.49 | 3.49 |  |  |  |  |  |
|  | APOBEC3A | 7.29 | 6.55 | 3.44 |  |  |  |  |  |  |  |  |  |  |
