# Supplementary material for: Molecular signatures of maturing dendritic cells: implications for testing the quality of dendritic cell therapies
Source: J Transl Med. 2010 Jan 15;8:4. doi: 10.1186/1479-5876-8-4 (PMC2841589; doi:10.1186/1479-5876-8-4)
Supplement: Additional file 3 — Table S3. Immature DC Genes whose expression was down-regulated following LPS and IFN- γ stimulation. The specific genes that were differentially expressed among the DCs stimulated with LPS and IFN- γ for different durations of time and their fold-change, down-regulated genes summary. (t-test, p ≤ 0.001 compared to hr 0). [file 1479-5876-8-4-S3.DOC]

**Table S3. Immature DC Genes whose expression was down-regulated following LPS and IFN-γ stimulation**

|  | **Gene** | **Gene expression fold decrease** | | |  | **Gene** | **Gene expression fold decrease** | | |  | **Gene** | **Gene expression fold change** | | |
| --- | --- | --- | --- | --- | --- | --- | --- | --- | --- | --- | --- | --- | --- | --- |
| **Category** | **Symbol** | **4 h** | **8 h** | **24h** | **Category** | **Symbol** | **4h** | **8h** | **24h** | **Category** | **symbol** | **4 h** | **8 h** | **24 h** |
| Chemokine | CCR1 | 3.61 | 2.84 | 2.73 | Antigen | HLA-DMA | 1.48 | 4.33 | 5.75 | Aminoacid | GATM | 2.70 | 11.98 | 17.67 |
| Receptors | CCR2 | 8.77 | 6.54 | 8.33 | Uptake & | HLA-DPA1 | NS | NS | 4.07 | Metabolism | SRM | 2.80 | 3.89 | 4.06 |
|  |  |  |  |  | Presentation | HLA-DPB1 | NS | 1.90 | 3.47 |  | GLUD1 | 2.27 | 3.09 | 2.40 |
| Cytokines & | IL17RB | NS | NS | 1.89 |  | HLA-DQA2 | NS | NS | 2.82 |  | RDH13 | 4.49 | 4.33 | 2.74 |
| Receptors | IL11RA | 2.01 | 1.84 | NS |  | HLA-DQB1 | NS | 2.02 | 4.43 |  | AMT | 2.77 | 3.29 | 1.63 |
|  | IL13RA1 | 1.56 | 1.93 | NS |  | HLA-DRA | NS | NS | 2.82 |  |  |  |  |  |
|  | IFNGR1 | 4.35 | NS | NS |  | HLA-DRB1 | NS | NS | 2.60 | Arachidonic | ALOX5 | 2.25 | 2.84 | 3.12 |
|  | IFNGR2 | 1.85 | NS | NS |  | HLA-DRB2 | NS | NS | 3.10 | Acid or | ALOX15 | 2.17 | 9.52 | 7.69 |
|  |  |  |  |  |  | HLA-DRB4 | NS | NS | 4.10 | Eicosanoid | LTA4H | 4.27 | 7.07 | 2.98 |
| TNF | TNFSF3 | 1.70 | 2.73 | 3.36 |  | HLA-DRB | NS | NS | 2.56 | Metabolism | PTGS1 | 5.43 | 20.41 | 12.99 |
| Superfamily | TNFSF12 | 5.26 | 8.40 | 5.76 |  | CDSIGN | 1.87 | 3.57 | 4.97 |  | TBXAS1 | 2.29 | 6.99 | 8.77 |
| & TNFR | TNFRSF1A | 1.86 | NS | NS |  |  |  |  |  |  | DHRS4 | 1.78 | 2.36 | 1.88 |
| Superfamily | TNFRSF3 | 1.80 | NS | NS | Endocytosis | AP2A2 | 8.57 | 11.6 | 7.32 |  | CERK | 12.3 | 9.81 | 5.26 |
|  | TNFRSF16 | 1.46 | 1.71 | NS |  | MRC1 | NS | 7.15 | 30.2 |  |  |  |  |  |
|  |  |  |  |  |  |  |  |  |  | Oxidative | UQCRC1 | 1.91 | 2.65 | 1.76 |
| TNF and | TNFAIP8L2 | 2.39 | 2.11 | 2.08 | c-type lectin | CLEC4A | 4.67 | 6.79 | 2.27 | Phosporyl- | NDUFB10 | 1.40 | 1.39 | 2.14 |
| TNFR | TRAP1 | 3.75 | 4.64 | NS | Superfamily | CLEC4G | 2.40 | 5.44 | 4.26 | Ation | NDUFS2 | 2.08 | 1.46 | 2.03 |
| Related | FAF1 | NS | 2.43 | 1.67 |  | CLEC10A | 3.82 | 28.2 | 43.5 |  | APT5D | 2.04 | 4.74 | 3.70 |
|  |  |  |  |  |  |  |  |  |  |  | ATP5A1 | 3.02 | 4.46 | 3.93 |
| TLR | SIGIRR | 2.27 | 2.50 | 2.00 | Vesicular | STX3 | 2.91 | 2.73 | 2.50 |  | COX5B | NS | 2.54 | 1.76 |
| signaling | LY96 | 1.45 | NS | NS | Transport | STX7 | 2.13 | 1.54 | NS |  | SDHA | 1.49 | 1.71 | 1.81 |
|  | IRAK1 | 1.95 | 2.01 | NS |  | STX10 | 4.19 | 4.70 | 3.50 |  |  |  |  |  |
|  | MAP3K7IP1 | 1.65 | 1.51 | NS |  | VAMP3 | 1.70 | 2..32 | 2.05 | Carbohydrate | HK3 | 1.83 | 2.72 | 9.17 |
|  |  |  |  |  |  |  |  |  |  | Metabolism | FBP1 | 1.89 | 7.26 | 7.69 |
| JAK-STAT | TYK2 | 2.34 | NS | NS | Growth | VEGFB | 3.12 | 5.54 | 2.99 |  | HEXB | 1.54 | 0.40 | 2.99 |
| Pathway |  |  |  |  | factors & | TGFBI | 2.44 | 6.76 | 11.2 |  | PMM1 | 2.93 | 3.69 | 2.44 |
|  |  |  |  |  | Receptors | TGFBR1 | 2.11 | 6.58 | 11.2 |  | PYGL | 2.22 | 3.34 | 2.80 |
| MAPK | DUSP3 | 3.67 | 3.39 | 2.33 |  |  |  |  |  |  | GALK1 | 2.37 | 3.64 | 1.90 |
| Signaling | RASGRP1 | 1.57 | 2.36 | 4.07 | Oncogenes | MYC | 9.17 | 8.83 | 9.35 |  | ACSS1 | 6.76 | 6.18 | 2.10 |
| Pathway | MAP3K3 | 4.06 | 3.60 | 2.04 |  | FOS | 2.45 | 2.71 | 2.97 |  | GPI | 1.75 | 2.91 | 2.00 |
|  | MAP3K5 | 1.76 | NS | NS |  | MAF | 15.0 | 8.30 | 20.5 |  |  |  |  |  |
|  | MAP3K6 | 1.99 | NS | NS |  | MAF1 | 1.50 | NS | NS | Ribosomal | RPL5 | 2.08 | 3.53 | 3.82 |
|  | MAP2K5 | 2.78 | 2.30 | NS |  |  |  |  |  | Proteins | RPSA | NS | 3.19 | 3.16 |
|  | MAPK9 | 3.10 | 2.47 | NS | Cell cycle | RASSF1 | 8.97 | 7.11 | 2.39 |  | RPS3A | 1.65 | 2.41 | 3.07 |
|  |  |  |  |  |  | CCNH | 3.20 | 2.53 | 2.52 |  | RPL7 | 1.72 | 2.41 | 2.98 |
| Activation | MAF | 15.02 | 8.30 | 20.5 |  | CCNY | 1.83 | 1.91 | 2.00 |  | RPS8 | NS | 3.42 | 2.97 |
| Protein-1 | MAFB | 2.47 | 2.98 | 3.56 |  | CCNG1 | 2.99 | 2.62 | NS |  |  |  |  |  |
|  | MAF1 | 1.50 | NS | NS |  | CDK2AP1 | 2.33 | 3.89 | 3.69 | Polymerase | POLD1 | 3.06 | 3.44 | 2.29 |
|  |  |  |  |  |  |  |  |  |  |  | POLD2 | 2.62 | 4.88 | 3.28 |
| Apoptosis | NAIP | NS | NS | 2.47 | G-protein | RGS10 | NS | 2.75 | 3.25 |  | POLE4 | 1.43 | 2.40 | 1.95 |
| Related | CASP6 | 2.33 | 2.12 | 1.70 | signalling | RGS19 | 9.48 | 5.09 | 3.81 |  | POLR1D | 1.93 | 1.99 | 1.46 |
|  | CIDEB | 1.43 | 1.48 | NS |  |  |  |  |  |  |  |  |  |  |
|  | DFFA | 1.49 | 1.43 | NS | Regulation | CEBPA | 15.1 | 15.1 | 5.84 | Others | ARHGDIB | 2.37 | 10.1 | 15.4 |
|  | DFFB | 2.22 | 1.90 | 1.67 | of transcription | NFE2 | 9.68 | 6.40 | 4.63 |  | DOK2 | 8.78 | 7.03 | 9.63 |
|  | NLRC4 | 2.51 | 2.25 | 2.34 |  |  |  |  |  |  | PPM1M | 8.99 | 8.72 | 3.97 |
|  | NLRP1 | 2.81 | 2.25 | NS | Purine & | RRM1 | 2.11 | 2.25 | 1.79 |  | VCL | 11.9 | 13.9 | 4.18 |
|  |  |  |  |  | Pyrimidine | DUT | 2.54 | 3.19 | 2.01 |  | CTSC | 13.0 | 1.38 | 6.74 |
| CDMarkers | CD1B | NS | 2.69 | 6.59 | Metabolism | DPYD | 3.36 | 5.68 | 3.57 |  |  |  |  |  |
|  | CD1C | 64 | 82.2 | 57.8 |  | TK2 | 3.97 | 3.77 | 2.98 |  |  |  |  |  |
|  | CD33 | 64.0 | 6.64 | 6.87 |  |  |  |  |  |  |  |  |  |  |
|  | CD14 | 1.48 | 1.66 | 1.96 |  |  |  |  |  |  |  |  |  |  |
