# Supplementary material for: Molecular signatures of maturing dendritic cells: implications for testing the quality of dendritic cell therapies
Source: J Transl Med. 2010 Jan 15;8:4. doi: 10.1186/1479-5876-8-4 (PMC2841589; doi:10.1186/1479-5876-8-4)
Supplement: Additional file 4 — Table S4. Soluble factor levels in DC cell culture supernatant whose expression was up-regulated following LPS and IFN- γ stimulation. Soluble factor levels in DC cell culture supernatant whose expression was up-regulated following LPS and IFN- γ stimulation. [file 1479-5876-8-4-S4.DOC]

| **Soluble Factors** | | **Factor Levels (pg/ml)** | |
| --- | --- | --- | --- |
|  |  | **Immature DCs** | **Mature DCs** |
|  | **GROa(CXCL1)** | 1.12 ± 0.41 | 45,720 ± 27,335 |
|  | **MCP1(CCL2)** | 23.6 ± 7.22 | 114.315 ± 161,556 |
|  | **IL6R** | 26.6 ± 15.73 | 1,143 ± 454 |
|  | **TNFRI(TNFRSF1A)** | 201 ± 19.4 | 722.32 ±237.09 |
|  | **IL16** | 114 ± 46.1 | 514 ± 111 |
|  | **TARC(CCL17)** | 240 ± 223 | 18,607 ±14,500 |
|  | **MDC(CCL22)** | 732.32 ±534.51 | 162,039 ± 177,267 |
|  | **MIP1b(CCL4)** | 15.08 ±8.65 | 81,493 ± 130,478 |
|  | **MIP1a(CCL3)** | 16.0 ± 8.03 | 69,426 ± 120,166 |
|  | **IL2** | 0.72 ± 0.39 | 48.7± 54.2 |
|  | **IL1b** | 1.08 ± 0.91 | 2,796 ± 4,827 |
|  | **MCP2(CCL8)** | 8.56 ± 4.05 | 910 ± 671 |
|  | **IL5** | 1 ± 0 | 9.28 ± 5.47 |
|  | **IL1a** | 0.64 ± 0.36 | 226 ± 185 |
|  | **ICAM1** | 28,525 ± 3,745 | 49,834 ± 8,490 |
|  | **Eotaxin(CCL11)** | 4 ± 1.1 | 16.2 ± 2.75 |
|  | **IL15** | 0.68 ± 0.33 | 9.36 ± 4.53 |
|  | **IL23** | 7.52 ± 9.58 | 1,561 ± 997 |
|  | **TGFa** | 8.36 ± 4.68 | 374 ± 193 |
|  | **TNFa** | 5.6 ± 4.34 | 4,843 ± 5,381 |
|  | **IL1Ra** | 754 ± 625 | 264,937 ± 198,263 |
|  | **IL8** | 13.1 ± 10.4 | 515,199 ± 233,905 |
|  | **MMP10** | 25.3 ± 10.8 | 18,736 ± 11,629 |
|  | **TNFRII(TNFRSF1B)** | 182 ± 28.0 | 4,929 ± 1,945 |
|  | **IL6** | 3.56 ± 2.43 | 302,731 ± 317,115 |
|  | **TIMP1** | 22,085 ± 7,797 | 1,887,200 ± 1,634,351 |
|  | **TIMP2** | 17,029 ± 2,367 | 27,484 ± 10,578 |
|  | **IL10** | 1.12 ± 0.27 | 1,561 ± 1,117 |
|  | **IL12p40** | 1.0 ± 0 | 10,056 ± 12,462 |
|  | **IL2R** | 140 ± 16.8 | 2,367 ±1,767 |
|  | **IL12p70** | 0.28 ± 0.11 | 155 ±162 |
|  | **IL13** | 0.28 ± 0.11 | 12.3 ± 11.8 |
|  | **RANTES9CCL5)** | 225 ± 129 | 2,903 ± 2,044 |
|  | **IFNa** | 4.08 ± 2.12 | 1.08 ± 0.3 |
|  | **ITAC(CXCL11)** | 0.64 ± 0.33 | 31.0 ± 59.3 |
|  | **IP10(CXCL10)** | 4.96 ± 5.62 | 2,805 ± 4,466 |
|  | **MIG(CXCL9)** | 30.6 ± 29.6 | 34,830 ± 61,285 |

**Table S4. Soluble factor levels in DC cell culture supernatant whose expression was up-regulated following LPS and IFN-γ stimulation**
