# Supplementary material for: Molecular signatures of maturing dendritic cells: implications for testing the quality of dendritic cell therapies
Source: J Transl Med. 2010 Jan 15;8:4. doi: 10.1186/1479-5876-8-4 (PMC2841589; doi:10.1186/1479-5876-8-4)
Supplement: Additional file 5 — Table S5. Soluble factor levels and fold changes in mature DC culture supernatant after 24 hours of CD40 Ligand stimulation. Soluble factor levels and fold changes in mature DC culture supernatant after 24 hours of CD40 Ligand stimulation. [file 1479-5876-8-4-S5.DOC]

**Table S5. Soluble factor levels and fold changes in mature DC culture supernatant after 24 hours of CD40 Ligand stimulation.**

| **Soluble Factors** | **Factor Levels (pg/ml)** | |  |
| --- | --- | --- | --- |
|  | **NonStimulated 24h** | **Stimulated 24h** | **Fold change** |
| **IL10** | 30.8 ± 28.17 | 1443 ± 1311 | 46.8 |
| **IL12p70** | 1.48 ± 1.17 | 49.4 ±6 5.64 | 33.4 |
| **IL6** | 27,980 ± 16,908 | 768,555 ± 686,628 | 27.5 |
| **TNFa** | 479 ± 415 | 12,569 ± 9,192 | 26.2 |
| **IFNg** | 21.4 ± 8.96 | 517 ± 986 | 24.2 |
| **IL23** | 48.2 ± 64.9 | 1,133 ± 740 | 23.5 |
| **IL12p40** | 119 ± 106 | 2,534 ± 1,526 | 21.2 |
| **COX2** | 1 ± 0 | 10.6 ±14.8 | 10.6 |
| **IL1b** | 900 ± 920 | 9,331 ± 12,175 | 10.4 |
| **Epiregulin** | 8.04 ± 7.97 | 69.6 ± 63.2 | 8.66 |
| **IL2R** | 1,197 ± 419 | 7,708 ± 5,558 | 6.44 |
| **IL13** | 1.28 ± 0.92 | 7.72 ± 5.96 | 6.03 |
| **RANTES(CCL5)** | 2,155 ± 1,403 | 10,477 ± 4,281 | 4.86 |
| **ITAC(CXCL11)** | 8.24 ± 2.8 | 37.1 ± 22.4 | 4.50 |
| **IP10(CXCL10)** | 884 ± 267 | 3,625 ± 1,995 | 4.10 |
| **MIP1b(CCL4)** | 37,670 ± 34,996 | 120,700 ± 201,197 | 3.20 |
| **MIP1a(CCL3)** | 57,711 ± 59,203 | 174,296 ± 176,091 | 3.02 |
| **MIG(CXCL9)** | 29,274 ± 27,806 | 63,980±47,243 | 2.19 |
| **TARC(CCL17)** | 22,868 ± 15,076 | 45,412 ± 43,619 | 1.99 |
| **IL1a** | 526 ± 591 | 1006 ± 813 | 1.91 |
| **IFNa** | 1.0 ± 0 | 1.8 ± 1.17 | 1.80 |
| **Eotaxin(CCL11)** | 9.76 ± 4.53 | 17.0 ± 1.16 | 1.74 |
| **MMP10** | 8,133 ± 6,695 | 13,709 ± 15,712 | 1.69 |
| **IL8** | 37,6878 ± 31,7129 | 534,675 ± 491,670 | 1.42 |
| **IL4** | 3.0 ± 2.2 | 4.12 ± 3.21 | 1.37 |
| **IL5** | 4.76 ± 4.43 | 6.36 ± 1.24 | 1.34 |
| **TGFb1** | 3,807 ± 1,824 | 5,015 ± 707 | 1.32 |
| **PEDF** | 128,839 ± 60,052 | 166,082 ± 93,506 | 1.29 |
| **IL15** | 12.0 ± 3.55 | 15.4 ± 4.48 | 1.28 |
| **GROa** | 10,029 ± 6,246 | 12,597 ± 9,148 | 1.26 |
| **IL2** | 23.9 ± 5.98 | 28.6 ± 5.75 | 1.20 |
| **Clusterin** | 193,696 ± 46,563 | 212,566 ± 43,338 | 1.10 |
| **IL7** | 1.0 ± 0 | 1.08 ± 0.18 | 1.08 |
| **TNFRII** | 2,707 ± 1,966 | 2,774 ± 1,418 | 1.02 |
| **MCP2** | 610 ± 519 | 616.72±244.53 | 1.01 |
| **TSP1** | 1.0 ± 0 | 1.0 ± 0 | 1.00 |
| **ICAM1** | 64,662 ± 22,717 | 61,460 ± 12,866 | -1.05 |
| **TIMP1** | 608,187 ± 564,353 | 567,633 ± 444,905 | -1.08 |
| **A2Macroglobulin** | 176,098,740 ± 67,603,614 | 163,012,180 ± 43,542,299 | -1.08 |
| **VCAM** | 74,465 ± 8,954 | 65,045 ± 5,341 | -1.15 |
| **MDC(CCL22)** | 195,839 ± 203,515 | 160,667 ± 196,323 | -1.22 |
| **SDF1b(CXCL12)** | 3.88 ± 1.45 | 2.92 ± 0.58 | -1.33 |
| **TRAIL** | 43.0 ± 9.38 | 31.3 ± 7.93 | -1.37 |
| **TGFa** | 289 ±182 | 203 ±88.7 | -1.43 |
| **TIMP2** | 33,326 ±12,039 | 20,325 ± 6,968 | -1.64 |
| **IL16** | 1,274 ± 1,184 | 686 ± 618 | -1.85 |
| **IL1ra** | 296,856 ± 237,543 | 126,677 ± 63,488 | -2.33 |
| **MCP1(CCL2)** | 13,258 ± 16,905 | 4,963± 7,232 | -2.70 |
| **IL6R** | 1,900 ± 963 | 704 ± 230 | -2.70 |
| **PECAM1(CD31)** | 7,497 ± 3,177 | 2,290 ± 2,363 | -3.23 |
| **TNFRI** | 952 ± 490 | 284 ± 74.0 | -3.33 |
| **TGFb2** | 230 ± 82.8 | 63.4 ±13.34 | -3.70 |
